# Developing an early support mHealth intervention for women and partners after perinatal loss: A secondary study of the e-Perinatal Project

**DOI:** 10.1192/j.eurpsy.2025.1422

**Published:** 2025-08-26

**Authors:** P. de-Juan-Iglesias, S. Carretero, C. García-Terol, R. Company-Córdoba, F. J. Nieto-Casado, C. Barquero-Jiménez, L. Goossens, J. J. Gil-Cosano, E. Motrico

**Affiliations:** 1 Perinatal-IBIS Lab, Institute of Biomedicine of Seville (IBiS), Seville; 2 Department of Developmental Psychology and Education, Universidad de Sevilla; 3 Psychology, Loyola University, Sevile; 4 Hospital maternoinfantil Sant Joan de Déu, Barcelona; 5 Department of Health and Biomedical Sciences, Loyola University, Sevile, Spain

## Abstract

**Introduction:**

Perinatal loss is a prevalent health concern worldwide, with one in four pregnancies ending in loss. Spontaneous perinatal loss (i.e., miscarriage, stillbirth, and neonatal death) is an excruciating experience that becomes part of parental identity and represents a unique form of grief. Online interventions offer a cost-effective and feasible option for reducing symptoms of depression, anxiety, and grief in cases of perinatal loss. Involving stakeholders during the early stages of content design and development results in interventions that are more relevant and effective.

**Objectives:**

This study aims to co-design evidence-based online content for offering psychological support in the early stages after perinatal loss and to integrate this content into the e-Perinatal app (mHealth application for the prevention of Perinatal Mental Health Disorders). It involves collaboration with local stakeholders, including bereaved women, non-birthing partners, and health professionals, to ensure that the intervention meets the population’s needs and aligns with the local sociocultural context. This is a secondary study of the ERC Starting Grant ePerinatal project (101042139), funded by Banco Sabadell Foundation.

**Methods:**

This study employed a qualitative design to conduct a multi-stage co-design and development process (pre-design, generative and evaluative phases). Two online focus groups were conducted: 1) bereaved women and non-birthing partners (n = 9), and 2) healthcare professionals with experience in perinatal loss care (n = 12). Following the presentation of the app prototype, participants were asked to provide feedback on both the evidence-based content (micro-intervention contents and information provided) and the app design features. The qualitative data were analysed using thematic analysis with NVivo software, while sociodemographic data were analysed through descriptive analysis. All data were coded by two researchers.

**Results:**

The analysis process is ongoing, and the emerging themes and subthemes are being categorized into: 1) user-related (e.g., health status, previous experience with the public health system, social support), 2) program-related (e.g., intervention content, formal and informal social connectedness), 3) user experience (e.g., information architecture [length], content strategy [wording]), and 4) professional-related (e.g., factors influencing healthcare professionals’ recommendations of the app).

**Conclusions:**

The content developed for perinatal loss will be designed to align with local stakeholders’ expectations and be integrated into the e-Perinatal app. By offering accessible support for women and their partners, the app will aim to help manage the psychological reactions often experienced during this vulnerable period, including grief, stigma, and an increased risk to mental health, as it is implemented in routine maternal care.

**Disclosure of Interest:**

None Declared

